# Adherence to anti-tuberculosis treatment among pulmonary tuberculosis patients: a qualitative and quantitative study

**DOI:** 10.1186/1472-6963-9-169

**Published:** 2009-09-18

**Authors:** Weiguo Xu, Wei Lu, Yang Zhou, Limei Zhu, Hongbing Shen, Jianming Wang

**Affiliations:** 1 Jiangsu Provincial Center for Disease Prevention and Control, Nanjing, PR China; 2 Department of Epidemiology and Biostatistics, School of Public Health, Nanjing Medical University, Nanjing, PR China

## Abstract

**Background:**

Tuberculosis (TB) patients have difficulty following a long-term treatment regimen. Efforts to improve treatment outcomes require better understanding of adherence as a complex behavioral issue and of the particular barriers to and facilitators of patient adherence.

**Methods:**

This study was carried out in Jiangsu Province of China with both quantitative and qualitative approaches. For the quantitative study, 780 sputum-smear positive TB patients consecutively registered since 2006 in 13 counties (districts) were queried with a structured questionnaire. Patients who had missed 10% of their total prescribed doses of TB drugs were deemed as non-adherent. Risks for non-adherence were estimated by computing odds ratios (ORs) and their 95% confidence intervals (95% CIs) using a logistic regression model. We also invited 20 TB patients and 10 local health workers for in-depth interviews. We then used content analysis based on this qualitative study to explore factors associated with non-adherence.

**Results:**

The proportion of non-adherence among 670 patients was 12.2%. Univariate analysis showed that patients, who were illiterate, divorced/widowed, lacked health insurance and were migrants, were more likely to be non-adherent. The crude ORs(95%CIs) were 2.38(1.37-4.13), 2.42(1.30-4.52), 1.89(1.07-3.32) and 1.98(1.03-3.83), respectively. The risk of non-adherence was lower among patients whose treatment was given under direct observation by village doctors or regular home visits by health workers, with ORs (95% CIs) of 0.19(0.10-0.36) and 0.23(0.10-0.51), respectively. In multivariate analysis, factors associated with non-adherence included illiteracy (OR: 2.42; 95% CI: 1.25-4.67) and direct observation by village doctors (OR: 0.23; 95% CI: 0.11-0.45). The in-depth interviews indicated that financial burdens and extra medical expenditures, adverse drug reactions, and social stigma were additional potential factors accounted for non-adherence.

**Conclusion:**

More importance should be given to treatment adherence under the current TB control program. Heavy financial burdens, lack of social support, adverse drug reactions and personal factors are associated with non-adherence. Direct observation and regular home visits by health workers appear to reduce the risk of non-adherence. More patient-centered interventions and greater attention to structural barriers are needed to improve treatment adherence.

## Background

Despite the recent progress of global efforts, tuberculosis (TB) is still one of the leading causes of morbidity and mortality world-wide, and remains as a major public health burden in many developing countries [1]. Current anti-tuberculosis therapy consists of a cocktail of drugs taken over a period of at least 6 months for new patients and 8 months for retreatment patients. Because of the long duration of the therapy, there is a risk of treatment interruption or default, a phenomenon that contributes to prolonged infectiousness, drug resistance, relapse and death [2,3]. The difficulty experienced by patients in following treatment regimens has raised the awareness of adherence as a complex behavioral issue [4]. Efforts to improve treatment outcomes require a better understanding of particular barriers to and facilitators of patient's adherence [5].

Studies of socioeconomic and behavioral factors affecting adherence have been conducted previously [6-9]. In Hong Kong, China, a study of 102 defaulters matched to 306 controls indicated that tobacco smoking, a history of prior treatment default or poor adherence, treatment side effects, and subsequent hospitalization were associated with treatment default [10]. A study in Fujian, China, that combined quantitative and qualitative methods reported that treatment adherence was associated with the intention of patients and the behavior of health service providers, but not with gender, age, career, education level or social stigma [11]. Another study in Chongqing, China, which involved interviewing patients and health staff, indicated that additional tests and drugs, especially liver protection drugs, may entail considerable financial barriers to starting and continuing treatment [7]. However, these aspects of treatment adherence have not been studied previously in Jiangsu Province of China. Therefore, to identify the social context, patient characteristics, and health system factors affecting patient's adherence to anti-tuberculosis treatment in Jiangsu Province, we conducted a study using both qualitative and quantitative methods. Our goal was to provide policy-makers with recommendation for more organized TB control program to improve the adherence to anti-tuberculosis treatment.

## Methods

### Study sites

This study was carried out in Jiangsu Province, which is located along the eastern coast of China and covers an area of 102.6 thousand square kilometers, about 1% of the total area of the country. Jiangsu Province contains 13 municipalities and 106 counties (districts), with a total population of 74 million. The population density is 736 per square kilometer, the highest among all provinces of China. The annual net per capita income of farmers in Jiangsu Province is 6561 Yuan ($964), and the annual employee salary is 27234 Yuan ($4005). The average life expectancy of the local population is 75.3 years, with men at 72.9 and women at 77.9 years, respectively. DOTS (direct observed therapy, short course) strategy for TB was introduced in the 1990s and is now 100% available at the county level. An internet-based surveillance system was set up in 2004, and all newly diagnosed TB cases are required to be registered with the local TB dispensary and reported to upper level health authorities.

### Data collection

This study was designed to use both quantitative and qualitative methods in order to gain insights into the factors that could contribute to adherence. A multi-stage sampling strategy was implemented for the quantitative aspects of the study. We selected 13 municipalities as the first sampling unit. In each municipality, one county (district) was randomly selected as the study site. In each site, 60 sputum-smear-positive TB patients registered since 2006 were consecutively selected as study subjects. The total estimated sample size was 780, given a confidence interval = 95%, an estimated non-adherent proportion = 20%, a relative precision = 0.2P, design efficiency = 2, and the number of study sites = 13. After obtaining informed consent, trained local TB dispensary staff interviewed all participants with a structured questionnaire, including basic characteristics, socioeconomic status, treatment history and adherence to anti-tuberculosis treatment. Non-adherent patients were further presented with 16 options for reasons of their non-adherence. In this study, observed treatment was divided into four categories: self-administered (patient took drugs without external observation); observed by family members or others (patient took drugs under the observation by family members or other volunteers); home-based drug delivery (local health workers sent anti-tuberculosis drugs to patients' home regularly, but did not observe them taking each dose of drugs); and directly observed by village doctors (patients took drugs under the direct observation by village doctors each time).

For the qualitative study, 20 TB patients (from Xuanwu and Taichang, 15 men, 5 women; 18 newly treated, 2 previously treated) were invited for in-depth interviews based on a convenience sampling strategy. A semi-schematized guide covering general as well as specific questions was utilized. The in-depth interview focused on patients' health-care seeking history, knowledge and attitudes towards TB, and their adherence to TB treatment. Ten local health workers from Taichang, including 5 village doctors and 5 community hospital doctors, were also invited for in-depth interviews. Themes for the in-depth interviews with these doctors included the current TB control program in the village/community, the main problems with and suggestions for the current TB program, treatment adherence of patients in the corresponding village/community, and the main reasons for non-adherence. Interviews were conducted in local health facilities by trained professionals from Jiangsu Provincial Center for Disease Prevention and Control with Chinese Mandarin. They lasted 20-40 minutes and were tape-recorded with permission.

### Data analysis

In this study, patients who had missed 10% or more of the total prescribed dose of TB drugs were deemed as non-adherent. For the quantitative study, data were entered in Epidata (Denmark) and analyzed using STATA 10.0 (College Station, TX, USA). Associations between selected factors and non-adherence were estimated by computing odds ratios (ORs) and their 95% confidence intervals (CIs) from an unconditional logistic regression model. Predictive variables that were independently significantly associated with treatment completion in univariate analysis were included in a multiple logistic regression model to determine their relative contributions in predicting treatment adherence while simultaneously adjusting for each of their effects. The criterion for significance was set at P < 0.05 based on a two-sided test. Continuous variables, including age and income, were converted to dichotomous variables using the median as the cutoff point.

For the qualitative study, content analysis was applied [12,13]. Tape-recorded in-depth interviews were firstly transcribed in Chinese characters and then translated into English by two trained staffs in the Jiangsu Provincial Center for Disease Prevention and Control. Codes were then developed based on the original terms used by participants. The transcripts and notes were analyzed thematically by categorizing the interview data under the main topic headings. The codes were then presented, discussed and checked within the research team. Tentative categories and sub-categories were created from the clustered codes, and subsequently main themes emerged based on the patterns and relationship between the categories.

### Ethical consideration

This study was approved by Ethics Committee in Center for Disease Control and Prevention. Oral informed consent was obtained from all participants.

## Results

### Findings from the quantitative study

A total of 670 patients were successfully enrolled and investigated in the study, with a response rate of 85.9%. The main reasons for loss to follow up were migration, unemployment, address or telephone number change, death and refusal to join the study. We compared some of the key characteristics of the 670 TB patients included in the analyses with those of 110 patients lost to follow up, and we found little difference in gender (73.4% and 73.0% were men, respectively) or age (51.4 years vs. 49.4 years, respectively). The average age (± s.d.) of study subjects was 51.4 ± 19.5 years old (men: 53.9 ± 18.9; women: 44.4 ± 19.5). Of these, 578 (86.3%) were new patients and 92 (13.7%) were retreated. The non-adherent proportion overall was 12.2% (82/670), which was similar between new patients and previously treated patients (12.6% vs. 9.8%). Approximately half of non-adherent patients interrupted their treatment intermittently (missed a total of more than 10% of all doses) and 41 stopped treatment before receiving 90% of their prescribed course. As shown in table 1, only 47.8% were treated with direct observation by village doctors and 16.9% took drugs without external supervision. Univariate analysis showed that patients, who were illiterate, divorced/widowed, lacked health insurance or were migrants, were more likely to be non-adherent. The crude ORs (95%CIs) were 2.38(1.37-4.13), 2.42(1.30-4.52), 1.89(1.07-3.32) and 1.98(1.03-3.83), respectively. The risk of non-adherence was lower among patients whose treatment was given under direct observation by village doctors or home visits by health workers, with ORs(95%CIs) of 0.19(0.10-0.36) and 0.23(0.10-0.51), respectively. In multivariate analysis, factors associated with non-adherence included illiteracy (OR: 2.42; 95% CI: 1.25-4.67) and direct observation by village doctors (OR: 0.23; 95% CI: 0.11-0.45) (table 2). The main reasons for non-adherence listed by patients (table 3) were adverse reactions to anti-tuberculosis drugs (37.8%), relieved symptoms (26.8%), long course regimen and large dose of drugs (15.9%), worry about dangers of drugs (15.9%), other disorders (15.9%), financial burden and medical expenditures (15.9%).

**Table 1:** Univariate analysis of association between selected factors and treatment adherence

**Variables**	**Total**	**Adherent**		**OR(95% CI)**	***P *value**
				
	**n**	**Yes, n(%)**	**No, n(%)**		
**Gender**					
Men	492	434(88.21)	58(11.79)	1.00	
Women	178	154(86.52)	24(13.48)	1.17(0.70-1.94)	0.555
**Age (years)**					
<53	326	292(89.57)	34(10.43)	1.00	
≥53	344	296(86.05)	48(13.95)	1.39(0.87-2.22)	0.165
**Education**					
Junior high school or over	325	295(90.77)	30(9.23)	1.00	
Elementary school	196	173(88.27)	23(11.73)	1.31(0.74-2.32)	0.361
Illiterate	149	120(80.54)	29(19.46)	2.38(1.37-4.13)	0.002
**Marital status**					
Married	495	441(89.09)	54(10.91)	1.00	
Unmarried	105	93(88.57)	12(11.43)	1.05(0.54-2.05)	0.877
Divorced/widowed	70	54(77.14)	16(22.86)	2.42(1.30-4.52)	0.006
**Family monthly income per capita (Yuan)**					
<450	328	288(87.80)	40(12.20)	1.00	
≥450	342	300(87.72)	42(12.28)	1.01(0.63-1.60)	0.973
**Health insurance**					
Yes	570	507(88.95)	63(11.05)	1.00	
No	100	81(81.00)	19(19.00)	1.89(1.07-3.32)	0.027
**Residence status**					
Permanent residents	606	537(88.61)	69(11.39)	1.00	
Migrants	64	51(79.69)	13(20.31)	1.98(1.03-3.83)	0.041
**Treatment history**					
Newly treated	578	505(87.37)	73(12.63)	1.00	
Retreated	92	83(90.22)	9(9.78)	0.75(0.36-1.56)	0.440
**Observed treatment**					
Self-administered	113	86(76.11)	27(23.89)	1.00	
Observed by family members or others	117	95(81.20)	22(18.80)	0.74(0.39-1.39)	0.347
Home-based drug delivery	120	105(87.50)	15(12.50)	0.46(0.23-0.91)	0.026
Directly observed by village doctors	320	302(94.38)	18(5.63)	0.19(0.10-0.36)	< 0.001
**Regular home-visiting by health workers**					
No	28	18(64.29)	10(35.71)	1.00	
Yes	642	570(88.79)	72(11.21)	0.23(0.10-0.51)	< 0.001

**Table 2:** Multiple logistic regression model to determine the factors predicting treatment non-adherence

**Variables**	**OR(95% CI)**	***P *value**
**Education**		
Junior high school or over	1.00	
Elementary school	1.32(0.70-2.50)	0.392
Illiterate	2.42(1.25-4.67)	0.008
**Marital status**		
Married	1.00	
Unmarried	1.18(0.56-2.51)	0.661
Divorced/widowed	1.84(0.93-3.64)	0.080
**Health insurance**		
Yes	1.00	
No	1.48(0.70-3.14)	0.308
**Residence status**		
Permanent residents	1.00	
Migrants	1.34(0.56-3.20)	0.508
**Observed treatment**		
Self-administered	1.00	
Observed by family members or others	0.76(0.38-1.50)	0.426
Home-based drug delivery	0.52(0.25-1.10)	0.086
Directly observed by village doctors	0.23(0.11-0.45)	< 0.001
**Regular home-visiting by health workers**		
No	1.00	
Yes	0.45(0.18-1.11)	0.083

**Table 3:** Factors associated with non-adherence listed by patients

**Reasons for non-adherence**	n*	**%**
1. Symptoms have been relieved and it is not necessary to continue treatment	22	26.83
2. Disease conditions have not been alleviated after treatment and the drugs seem to be ineffective	6	7.32
3. Treatment is not necessary as I am so old	7	8.54
4. I am working busy	5	6.10
5. Treatment course is too long and the dose is too large	13	15.85
6. I always forget to take drugs	11	13.41
7. Adverse drug reactions are severe	31	37.80
8. I worry about my body damaged by anti-tuberculosis drugs	13	15.85
9. Appetite is influenced after taking drugs	8	9.76
10. Other diseases cause interruption	13	15.85
11. Migration	8	9.76
12. Following doctor's advices	9	10.98
13. Following other's suggestions	4	4.88
14. I am not satisfied with health-care services	2	2.44
15. Financial difficulty and higher medical cost	13	15.85
16. Other reasons	19	23.17

*: Total number of non-adherent patients = 82

### Findings from the qualitative study

A total of 19 patients (95%) and 9 doctors (90%) were successfully interviewed. The main findings from the in-depth interviews are as follows:

#### (1) TB services are not actually free

Under the current "free service policy", patients report that they still need to pay extra fees for medical examinations, hospitalizations, and liver protection drugs. The additional cost is regarded as an important issue related to treatment adherence and varies from hundreds to thousands of Yuan per month. This sum accounts for a large part of the usual family income (the annual net capita income of farmers was 6561 Yuan and the annual employee's salary was 27234 Yuan in 2007). Drugs thought to protect the liver are frequently prescribed as part of treatment for TB and are major contributors to overall costs. These supposed liver protection drugs include herbal preparations, finished manufactured herbal products, combinations of vitamins and other non-herbal substances, and pharmaceutical preparations, most of which are not free of charge under the current "free service policy". The larger and more specialized health facility that patients visit, the higher the cost of the liver protection drugs is. Patients treated in urban specialized TB dispensaries are usually recommended by their doctors to be hospitalized, a process that entails much more cost than being treated at rural dispensaries.

"My spouse newly passed away and I also lost my job. To survive, I have to take drugs. Though anti-tuberculosis drugs are free of charge, other medical tests and liver protection drugs still cost me up to 2,000 Yuan. It is a heavy burden on me." (Patient)

Free anti-tuberculosis drugs provided by the local government are limited to the specified brands. Many patients complain about adverse reactions and the low quality of these drugs. Although it is only hearsay that free drugs are worse than privately purchased drugs, such information is widespread among patients in many sites. Local doctors also admitted that they sometimes recommend to patients, especially those who are old and weak, that they buy drugs rather than use the freely provided drugs. Patients with drug resistant infections, especially those with multi-drug resistant TB, face much greater difficulties since the second line anti-tuberculosis drugs are not free under the current national TB program. Both patients and local doctors reported that treatment is sometimes discontinued because of the higher financial burden of these drugs.

"When I was hospitalized, the doctor asked me to make a choice between free drugs and private expense ones. I knew nothing about their difference. The doctor recommended me to buy private expense drugs instead of using free ones, because the latter are worse. Afterward, other doctors told me the truth that both are the same. It cost me more than 10,000 Yuan for the first month treatment." (Patient)

"One patient aged 30 years old, coming from Shanxi Province, had good adherence at the first stage. However, after five months of treatment, the sputum smear test was still positive. I recommended him to do drug susceptibility test. Unfortunately, he was defined as a multi-drug resistant patient. Doctors in Shanghai Pulmonary Hospital recommended him to be hospitalized for standard treatment. Finally he gave up as the estimated cost was up to 10,000 Yuan. Can you imagine that his whole income per month was only 1,000 Yuan? The only thing he could do was giving up the treatment." (Doctor)

#### (2) Adverse reactions

In-depth interviews among both TB patients and local doctors indicate that adverse drug reaction is a reason for treatment non-adherence. Fear of the risks of adverse drug reactions leads some TB patients to interrupt treatment. Local health workers often cannot detect this discontinuation of treatment due to the lack of an active adverse drug reaction surveillance system under the current DOTS program.

"The majority of TB patients in my village have good adherence to treatment. However, some patients are reluctant to cooperate with us. The main reasons are the adverse reactions and long course of treatment. For example, one patient didn't visit my clinic to take drugs as regularly. So I called him immediately. He told me that he didn't want to continue as he felt much more uncomfortable after taking drugs." (Doctor)

#### (3) Stigma

A stigma attached to TB in China can lead to imposition of socio-physical distance and participatory restrictions on those suffering from the disease. The stigmatizing attitudes and behaviors of the community members towards the disease and its sufferers may lead individuals with TB to hide the diagnosis from others and to default from treatment. Although the National TB Program recommends that patients be treated under direct observation, some patients prefer to take their drugs at home without supervision. One reason that they do this is to conceal the disease from others due to fear of being discriminated against and isolated. Patients who are employed risk being fired if the employer knows they have TB. Unemployment, and the high cost of disease, can put heavy financial burdens on the family and lead patients to terminate treatment. Some migrant workers with TB have to move because they are driven out by their landlords or dismissed from their factories after being diagnosed. Because of this, patients would like to keep their names and addresses secret, which make it difficult for local health workers to visit and follow them. This situation is much worse among migrant patients.

"I lost my job after being diagnosed with TB. Without income, I can't afford the treatment. Moreover, my daughter needs money to pay for the tuition fee and I need to repay the debt for my newly built house. Now I have owed the hospital hundreds of Yuan." (Patient)

"Nonlocal patients change phone numbers frequently. Addresses they present usually are inaccurate on purpose, which makes it difficult to follow. I think one of the main reasons is that they are afraid of stigma and losing job." (Doctor)

## Discussion

Early detection of patients and providing effective treatment are the main interventions to prevent the spread of TB. However, current long-term anti-tuberculosis therapy could easily lead to patient non-adherence, which presents an important barrier for TB control programs [8,14]. Patients' adherence to their medication regimens has been reported to be influenced by the interaction of number of factors [8]. These various factors may be grouped as: health-system factors, social and family factors, and personal factors [15]. The factors that influence patient adherence to TB treatment vary in different populations, and those that emerged in our current study might be similar to, or different from, those reported in other areas of China.

The financial burden on TB patients was one of the key issues we found to be associated with non-adherence in our study. Although the government of China has established a "free TB service policy" with the aim of decreasing the financial burden on patients, this free policy seems to be not as well known or well implemented as planned in some areas [12,16]. In a previously reported study in four provinces of China [17], TB patients were obliged to pay 12-40% of their annual income for TB services, despite the fact that all smear-positive and some severe smear-negative patients received free drugs. It is believed that the heavy financial burden on patients is one of the main reasons that some TB patients fail to access and complete the treatment. The main financial burdens, as evidenced in the present study, are the extra cost for medical examinations, the need to purchase liver protection drugs, and hospitalization costs.

Conflicts of interest may be an underlying reason for the existence of heavy financial burdens on patients in the presence of the free treatment policy. Briefly, with the transformation from a traditional planned economy to a market-oriented economy beginning in the early 1980s, a larger part of rural health facilities in China were either totally or partially privatized [18]. These rural facilities have developed a variety of means to attract patients, in an effort to generate more revenues by providing more clinical services and selling more drugs [18]. Even in the state-owned hospitals, a fee for service and bonus-related revenue system has been adopted to encourage medical staff to make more money, a process that can increase the economic burden on patients, elevate health-care costs, and ultimately impede effective TB control in China.

Drugs are frequently prescribed in some countries, and especially in China, to protect the liver during treatment for TB [19]. A systematic review based on 85 research articles, which evaluated 30 distinct types of putative liver protection compounds, found no reliable evidence to support prescription of drugs or herbs for this purpose to people on TB treatment [19]. However, a variety of presumed liver protection drugs are still widely used in China and their purchase accounts for a large part of patients' medical expenditures. On one hand, doctors prescribe these drugs with the hope that they will prevent hepatic injury induced by anti-tuberculosis treatment. On the other hand, doctors generate more revenue by selling these drugs, as liver protection drugs are not freely provided under the current TB program. To quote one doctor in our study, "if we only deliver free anti-tuberculosis drugs rather than selling private expense drugs to patients, how can we get the bonus?"

Adverse reactions to current TB treatment, including hepatic injury, also contribute to non-adherence, as has been described in numbers of studies [8]. Xia reviewed reports published on this topic between 1996-2005 in China and found that the overall incidence of anti-tuberculosis drug induced adverse reactions was 12.6%, with hepatic injury the most common one (mean = 11.90%, median = 15.66%, range 0.75%-71.43%) [20]. In our present study, adverse drug reaction to TB treatment was also listed by patients as the most common factor (37.8%) associated with non-adherence. Thus, active surveillance of patients' adverse reactions would be far preferable to merely prescribing possibly ineffective liver protection drugs. To minimize the impact of adverse reactions, it is important that health staff provide concise pretreatment counseling to patients and that they manage such side-effects with timely recommendations and services [21].

TB was originally named "Phthisis" and regarded as a deadly disease in China. With the development of modern medicine, TB is no longer an incurable disease. However, TB patients still experience stigma and are always isolated from the community. Although the Chinese government has carried out massive education programs in recent years, knowledge of TB among the general population is still limited [16]. Social support can help patients overcome structural barriers as well as personal barriers, and community and family members' attitudes may influence a patient's decision whether to stop or continue TB treatment. In such circumstances, community-based TB treatment programs and stronger involvement of local social networks to support TB patients may be justified [6].

Direct observation of treatment (DOT) has been recommended by WHO to enhance patients' adherence and is regarded as a main component of the "breakthrough" in TB control programs [22]. However, DOT is not implemented as well as expected in some areas of China [7,23,24]. A study in Chongqing reported that less than 5% of TB patients had been treated under direct observation by health staff, and 12.5% of patients reported "interrupted treatment" [7]. In Shandong Province, only 21% of patients took their medicine under the surveillance of village doctors, and this proportion varied widely across the counties, from 0% to 70% [23]. Our findings from this study also proved that DOT is not well accepted in some rural areas of Jiangsu. Other alternatives need to be studied as possible ways to reinforce adherence in patients who do not accept direct observation treatment in village clinics. In Jiangsu Province, patients usually visit the TB dispensary monthly and get the anti-tuberculosis drugs for the whole month. They swallow the drugs under the direct observation by doctors or family members or supervise by themselves. Due to different reasons, some patients might not attend the regular visits to TB dispensary, resulting in the interruption of treatment. In such a case, local health workers are responsible for delivering drugs to patients' home and perform patient retrieval. Strictly, it is not a real direct observation because health workers don't observe patients swallowing each dose of drugs. But it is still believed to be an alternative way to help increase the adherence, given that DOT is not accepted by the patient. A centerpiece of the DOTS strategy is that it shifts responsibility for completion of therapy and successful clinical outcomes to the healthcare provider rather than to the patients themselves [25]. It is clear that good health services are necessary, but not sufficient, to ensure treatment success [15]. Patients still need to choose to take drugs. Building supportive patient-doctor relationships, rather than solely relying on authoritarian supervision, can best improve treatment adherence and TB control [26].

Several methodological issues should be discussed. Firstly, results of this study come from Jiangsu Province and may not represent the conditions in all of China. Secondly, 14.1% of selected subjects could not be traced and were not involved in the analysis, raising the possibility of some selection bias. Thirdly, data on treatment history and possible explanatory factors in this study were based on self-report of patients. Recall bias and investigator bias were thus unavoidable.

## Conclusion

More importance should be given to treatment adherence under the current TB control program. Heavy financial burdens, lack of social support, adverse drug reactions and personal factors are associated with non-adherence. Direct observation and regular home visits by health workers appear to reduce the risk of non-adherence. More patient-centered interventions and greater attention to structural barriers are needed to improve treatment adherence.

## Competing interests

The authors declare that they have no competing interests.

## Authors' contributions

WX, WL, YZ and LZ conceived the idea and implemented the field study. WX, HS and JW participated in the statistical analysis and wrote the manuscript. All authors read and approved the final manuscript.

## Pre-publication history

The pre-publication history for this paper can be accessed here:


